# Valuable Medicine

**Published:** 1856-03

**Authors:** 


*Valuable Medicine.*—A Yankee doctor has contrived to extract from sausages a powerful tonic, which, he says, contains the whole strength of the original *bark;* he calls it the "Sulphate of Canine!" He anticipates a great popularity for it in New York city.—*Worcester Transcript.*

Dr. Gassem is the New York general agent for this beautiful medicine. He takes a smashing store on Broadway next week, and means to have the whole front covered with an allegorical painting of the "Canine" taking hydra-headed disease by the throat. He means to advertise in the largest dailies, as they say in the country, "to kill;" and will start two secular monthlies and one religious paper to write it up. The clergy he will supply gratuitously with bottles. He has engaged several Irishmen to meet severe accidents and be carried into his store for relief, and suborned the Police on that beat to inform the reporters of the fact. Dr. Gassem's wife will wear the largest hoop and the smallest bonnet in town. His carriage will attend a Fifth avenue church, and a law suit touching the right to vend the article will be entered in February. So it will be seen that the popular methods of getting the "Canine" before the public have all been secured. If any body has city lots to sell, the doctor will be a good customer a year hence.

